# Design Guidelines for Online Health Forums: User-Centered Design Approach

**DOI:** 10.2196/85411

**Published:** 2026-09-14

**Authors:** Paul Marshall, Neil Caton, Anna Lindroos Cermakova, Bohdana Dock, Zoe Glossop, Steven Jones, Jade Haines, Christopher Lodge, Karen Machin, Jo Rycroft-Malone, Rachel Meacock, Dorian Peters, Paul Rayson, Heather Robinson, Louisa Salhi, Jay Staff, Fiona Lobban

**Affiliations:** 1 Spectrum Centre for Mental Health Research, Division of Health Research, Lancaster University, Health Innovation One, Bailrigg, Lancaster, United Kingdom, 44 01524 65201; 2 Mental Health Innovations, London, United Kingdom; 3 IT Corporate Services, Berkshire Healthcare NHS Foundation Trust, Berkshire, United Kingdom; 4 Independent Researcher, Lancaster, United Kingdom; 5 Faculty of Health and Medicine, Lancaster University, Lancaster, United Kingdom; 6 Division of Population Health, Health Services Research and Primary Care, University of Manchester, Manchester, United Kingdom; 7 Dyson School of Design Engineering, Imperial College London, London, United Kingdom; 8 Leverhulme Centre for the Future of Intelligence, Institute for Technology and Humanity, University of Cambridge, Cambridge, United Kingdom; 9 School of Computing and Communications, Lancaster University, , Lancaster, United Kingdom; 10 Kooth Digital Health, London, United Kingdom

**Keywords:** digital mental health, peer-to-peer support, social networking, qualitative research, internet-based intervention, self-determination theory, user-centered design

## Abstract

**Background:**

Online health forums are used widely, yet evidence of their effectiveness is inconsistent. Evidence-based forum design guidance grounded in theory and lived experience could improve the efficacy and outcomes of these forums for the many people using them worldwide.

**Objective:**

This study aimed to draw on the experience of online forum users and staff, and insights from existing research on technology design and self-determination theory, to generate a set of theoretically grounded guidelines for safe and well-being–supportive online forums.

**Methods:**

We conducted 54 semistructured interviews (36 forum users and 18 forum staff) and 4 design workshops with forum staff, combined with input from a multidisciplinary research team. Principles of qualitative framework analysis were used to adapt a preexisting framework for well-being–supportive technology to the context of online health forums.

**Results:**

The resulting design guidelines are framed around 4 overarching principles relating to the psychological needs for autonomy, competence, and relatedness as defined by self-determination theory, and the additional need for safety in online forums. Each principle is presented alongside pragmatic design heuristics and specific implementation strategies.

**Conclusions:**

User experiences of online health forums are mixed. We have drawn on self-determination theory to propose evidence-informed guidelines for service development and refinement, adaptable to specific user groups across diverse settings.

## Introduction

Online forums are a popular medium for text-based communication. Also referred to as discussion boards or online communities, they allow users to create discussion threads and respond with subsequent posts [1]. Other users can review threads to access relevant discussions, facilitating asynchronous group interaction on community-generated topics. Most are managed by moderators who ensure safety, support users, and promote activity [2]. Forums typically allow free, anonymous, and 24-hour access, representing a flexible way to discuss shared interests and experiences.

There is a well-established literature on the impacts of online health forums. Reviews highlight how forums facilitate connection and the exchange of health management advice derived from personal experience [3-5]. Some users report the normalization of distressing health experiences, reduced isolation, and feeling empowered by supporting others [6]. Many forums are maintained by people with personal experience of health conditions using social media websites. Others are hosted on dedicated platforms run by health services, charities, or corporate providers. Moderation may therefore be provided by people with lived experience, trained volunteers, health professionals, or a team with a combination of these backgrounds [7]. Evidence of the effectiveness of health forums is mixed, with some trials reporting positive impacts on outcomes such as distress and self-efficacy, and others showing no significant impact on health outcomes [8-10]. Some users also report negative experiences, including exposure to distressing content and health misinformation [11,12].

Use of health forums is growing and is likely to expand as the need for mental health support continues to outstrip access to in-person services [13,14]. It is therefore vital that those seeking to establish and promote forums can access evidence-informed strategies to optimize their safety and effectiveness. Existing guidance recommends that forums should aim for sustainable growth by welcoming new users, supporting and rewarding core members, and developing content such as newsletters to promote community visibility [15]. Guidance for health professional moderation highlights the importance of skills for facilitating group engagement and validating users’ experiences [16]. However, theory-informed guidance that draws on the expertise of those with both personal and professional experience of health forums is absent in this literature. This is significant given Medical Research Council guidance emphasizing theory’s role in articulating how design decisions create impact, and the importance of stakeholder perspectives in ensuring intervention development is guided by real-world experience [17].

To address this, we report design guidance for health forums based on an existing technology design framework [18] informed by self-determination theory (SDT). SDT is a theory of motivation, personality, and well-being with a strong empirical basis spanning 4 decades across cultures and health domains [19]. The theory posits that well-being is underpinned by the satisfaction of basic psychological needs for autonomy (volition and alignment with personal values), competence (feeling capable and effective), and relatedness (connection and belonging) [20]. Our guidance builds on recent research using SDT to inform technology design, predicated on the notion that technology that promotes psychological needs will better support engagement and well-being [21,22]. Specifically, we adapted SDT-informed guidelines for well-being–supportive technology design by Peters [18], derived from patterns of contextual factors that predictably lead to psychological need satisfaction based on literature across domains (ie, health, psychology, coaching, and education), synthesized and then recontextualized for the technology domain.

The guidelines are presented as a hierarchical framework with 3 levels [18]. Principles of well-being–supportive design correspond to psychological needs (ie, technology should support user autonomy). Subordinate to these principles are heuristics, or practical rules of thumb for technology design (eg, “apply best practice for accessibility”). Subordinate strategies are specific implementation actions corresponding to each heuristic (eg, a strategy sitting within the heuristic “provide meaningful choices” is “offer customizable profiles”). We adapted these guidelines for the context of online health forums by integrating the perspectives of a range of stakeholders with experience and expertise in this domain. By offering actionable recommendations embedded within an established theoretical perspective, our goal is to allow forum designers and hosts across health settings to derive insights applicable to their diverse contexts.

## Methods

### Study Design

This paper is part of the Improving Peer Online Forums (iPOF) project [23], a realist study involving evidence synthesis to generate program theories regarding forum impacts [6,7], tested using mixed methods evaluation [24,25]. This paper translates data collected as part of this evaluation into actionable design guidance structured around SDT. The interdisciplinary iPOF team included academics working across psychology, linguistics, computer science, and health economics; lived experience experts; forum staff; and clinicians. The wider team included partner forums with representatives from statutory, corporate, and charity health services. While iPOF had a primary area of expertise in mental health, partner organizations also hosted forums on physical health, young people’s well-being, and carer support. A deidentified description of the partner organizations’ forums is on the study website [26]. We used a qualitative approach, integrating interview data and workshop discussions with a preexisting framework, before drawing on the multidisciplinary team’s expertise to refine the findings.

### Stage 1: Data Collection

We collected data using semistructured interviews and workshops. Interviews were conducted with forum hosts (n=18) including moderators (n=13) and senior community managers (n=3) working at organizations running health forums, in addition to academics within the iPOF team who had experience designing, implementing, and evaluating health forums in clinical settings (n=2). Staff were predominantly female (n=14) and White British (n=14), aged between 16 and 65 years. A second round of interviews (n=36) was conducted with users of our partners’ forums, sampled via adverts posted on the forums. These forums offered support for a range of health challenges including anxiety and depression, eating disorders, and young people’s well-being. Users were predominantly female (n=30) and White British (n=30), aged between 16 and 85 years. Full demographic details are provided in Multimedia Appendix 1.

Interviews were conducted between August 2023 and October 2024 either by PM or by Zoe Glossop via phone or Microsoft Teams, audio-recorded, and transcribed by a professional transcription service for analysis in NVivo (version 12; QSR International). As per the iPOF study aims, interviews sought to understand the underlying mechanisms explaining how forums impact users and moderators. All participants were asked about forum features and their refinement (eg, “What are the features of the forum?” “What do you like and what could change?” and “What advice would you give to forum designers?”). Results regarding forum impacts are reported elsewhere [6,7]. We conducted a secondary analysis of these interview data for this study, focused solely on insights relevant to forum design. Sample size was informed by the principle of information power [27], which proposes that studies with greater specificity in research focus and participant characteristics require fewer interviews. Given our focused aim of adapting preexisting guidelines to the narrower forum context, the research team ceased interviewing once sufficient data had been gathered to meaningfully adapt the framework.

We subsequently held four 2-hour design workshops on Microsoft Teams with a group of forum hosts separate from the core research team, who were either senior community managers or forum moderators. Three workshops separately explored the psychological needs for autonomy (n=4; 3 community managers and 1 moderator), competence (n=5; 3 community managers and 2 moderators), and relatedness (n=10; 7 community managers and 3 moderators). We held an additional workshop focused on designing forums to promote safety (n=8; 6 community managers and 2 moderators), which emerged as a priority area for designers, staff, and users in our team’s prior work [6,25]. Researchers facilitating these workshops first introduced each concept before inviting attendees to share ideas for their promotion (ie, “How can forums be designed to promote user autonomy?”) Researchers captured participants’ ideas using detailed field notes, which were added to NVivo 12 for analysis alongside interview data. Interview topic guides and interview and workshop participant demographic details are available in Multimedia Appendix 1.

### Stage 2: Coding a Draft Framework

Data analysis was informed by the principles of qualitative framework synthesis [28], an approach suited to adapting existing conceptual frameworks using novel data. This involves coding data both deductively (to existing concepts within the framework) and inductively (to generate new concepts where data do not fit) and refining the framework iteratively. We used Peters’ [18] technology design guidelines as the starting point for the analysis. As noted, the guidelines are presented as a hierarchical framework with 3 levels of granularity (principles, heuristics, and strategies).

At the outset of the analysis, we replicated Peters’ framework in NVivo 12, with each principle, heuristic, and strategy represented as a node to which interview and workshop data could be coded. PM and ZG conducted initial data analysis using a combination of deductive and inductive coding. Codes were regularly discussed in weekly team meetings to challenge and develop emerging insights. Our goal was to use these codes to populate the framework with content specifically related to online health forums. Deductive coding involved linking data extracts to preexisting strategies and heuristics. Where data did not relate to an aspect of the framework, they were coded inductively and grouped to create new heuristics and strategies.

Twelve of the original 15 heuristics were retained, with strategies modified to reflect forum design preferences described by participants in our workshops and interviews. However, as Peters notes “some will apply more readily than others for a given technology or project” [18]. Indeed, 3 heuristics not supported by our data were removed at the end of the coding process: “support optimal challenge” (relevant for games or technologies with advanced users but not to health forums), “provide nonevaluative feedback” (relevant for users of education and behavior change technologies), and “provide effectance-relevant feedback” (performance feedback is typically not given to health forums).

### Stage 3: Refinement Through Stakeholder Engagement

The draft framework was shared with and refined in response to written feedback from workshop participants and the wider iPOF team, including coauthors with personal experience of designing, moderating, using, and evaluating online mental health forums. Reflexivity refers to researchers’ awareness of the ways in which their views, experiences, and positionality influence the research process [29]. To enhance rigor, this analysis was developed in collaboration with a diverse team of researchers, health professionals, forum staff, and lived experience experts. We recognize the potential inhibiting effect of power dynamics between forum staff and users, which may have influenced disclosure within workshops and in the development of the manuscript. This was balanced by the use of confidential individual interviews conducted by university researchers external to the partner organizations. No forum user was interviewed by staff from their own forum.

### Ethical Considerations

Ethical approval for this research was granted by Solihull Research Ethics Committee (IRAS 314029). All interviewees provided written informed consent and were offered a £20 shopping voucher (£1=US $1.35 as of October 1, 2024). Interview data were anonymized and forum names pseudonymized to promote confidentiality.

## Results

### Overview

The resulting framework for the design of health forums comprises 4 principles framed around designing for autonomy, competence, relatedness, and safety. Principles are reported alongside 18 corresponding heuristics, each with specific implementation strategies. Principles and heuristics are presented in Textbox 1. Illustrative data extracts for each strategy are presented in Multimedia Appendix 2. It should be noted that implementation decisions may promote multiple psychological needs simultaneously. We have presented heuristics and strategies under the psychological need they most directly support.

Textbox 1. **Guidance for well-being–supportive design adapted for online health forums.**

Principle 1: Support autonomy

- Heuristic 1: Apply best practices for accessibility
- Heuristic 2: Empathize with your user’s frame of reference
- Heuristic 3: Provide meaningful rationale
- Heuristic 4: Provide meaningful choices
- Heuristic 5: Support informed choices
- Heuristic 6: Communicate in autonomy-supportive rather than controlling ways
- Heuristic 7: Ensure rewards are autonomy supportive
- Heuristic 8: Support mindful attention

Principle 2: Support competence

- Heuristic 9: Apply best practices for usability
- *Heuristic 10*: *Communicate guidelines and protocols*

Principle 3: Support relatedness

- Heuristic 11: Support a sense of meaningful connection to others
- Heuristic 12: Support a sense of belonging
- Heuristic 13: Support caring for others

Principle 4: Support safety

- *Heuristic 14*: *Establish community guidelines*
- *Heuristic 15: Establish procedures for managing distress*
- *Heuristic 16*: *Consider the limits and impacts of anonymity and confidentiality*
- *Heuristic 17*: *Manage content visibility*
- *Heuristic 18*: E*stablish procedures for managing problematic forum use*

Heuristics in italics represent those added to the original guidance framework for well-being–supportive technology design [18]. Heuristics not in italics represent those retained from the original framework.

### Principle 1: Support Autonomy

The psychological need for autonomy refers to the need to feel volitional self-endorsement of one’s own actions [30]. Autonomy is supported when users have ready access to and are well informed about the technology at hand [18]. This should allow users to make informed and meaningful decisions about how they engage with it in ways that are consistent with their personal goals and values.

#### Heuristic 1: Apply Best Practices for Accessibility

Ensure that online health forums are accessible by accounting for the different support needs and access formats of the potential user base.

##### Strategy 1.1: Conduct Accessibility Testing With Diverse Users and Formats

Health forums are accessed by people with a range of differing cognitive and physical abilities, using various devices including desktop computers, laptops, mobile phones, and tablets, and with different operating systems. Ensure that forum topics can be navigated intuitively on each device and by diverse users. Particular attention should be paid to the appearance of the forum on mobile web browsers, which may be more difficult to navigate given the small size of these devices. Within threads, posts should be ordered clearly and sequentially so that there is an easy-to-follow flow to online conversations. Integration with supportive technologies for use by those with additional access needs, such as screen readers, should be considered. See Web Content Accessibility Guidelines 2.2 for details on developing accessible digital services [31].

### Heuristic 2: Empathize With Your User’s Frame of Reference

Eliciting users’ perspectives on their goals for forum use increases the likelihood that the service meets their needs. Do so in a way that expresses a genuine desire to understand, learn, and act on their views.

#### Strategy 2.1: Use Co-Design Methods

Co-design refers to service development by multiple stakeholders, most often health service staff or researchers and those with lived experience. There is a growing body of work detailing how to conduct meaningful and genuinely collaborative co-design [32], including for digital health services [33-35]. Seeking diversity in user perspectives, including less frequent users and those with different backgrounds and experiences, is likely to promote forum accessibility among a wider audience of potential users. Such work should be appropriately resourced to allow for content to be regularly reviewed and refreshed as the community evolves.

#### Strategy 2.2: Seek Community Perspectives

Include a way for users to provide ongoing feedback on their forum experience. Options include a “report a bug” function, a specific “feedback” thread to which users can post about challenges or suggestions for updates, community polls or surveys, and via establishing a user advisory group. Consider using surveys to improve the representativeness and diversity of data on user perspectives. This can complement co-design methods, which are usually conducted with smaller groups and are more resource intensive. Surveys can be targeted for specific purposes, such as gaining perspectives on new forum features at the design phase, or feedback on recent changes. Forums should aim for an iterative and inclusive dialogue with forum users to shape the community in their interests [36]. A codeveloped theory of change identifying the way in which a forum is intended to operate can further guide data collection, identify areas for intervention optimization, and highlight both expected and unintended impacts.

### Heuristic 3: Provide Meaningful Rationale

To promote willing endorsement in user experience, explain why forums function as they do. Make rationales meaningful by using accessible language and include details relevant to the user experience, avoiding unnecessarily technical or abstract information.

#### Strategy 3.1: Articulate Reasons for Forum Functions and Limitations

Providing clear, accessible information regarding the scope and function of a forum can promote informed engagement and reduce the likelihood that users feel frustrated if they later find that the forum does not operate as expected. Such content could include details regarding the intended function of the forum and what the forum is not equipped to deliver, for example, clarifying that the forum is meant for peer support and not medical advice, if appropriate. This could be presented in a visible place within the forum, such as a sidebar, a post pinned to the top of the main page, or via an automatic response to their initial forum posts.

### Heuristic 4: Provide Meaningful Choices

Provide options for modifying the forum experience in line with users’ goals and values.

#### Strategy 4.1: Provide Personalization Options

Include personalization options likely to be valued by forum users. This could include the use of customizable profile pictures or virtual avatars, profile pages with space to include a user biography, or an editable signature that appears underneath users’ forum posts. This could serve to humanize users’ profiles and offer a point of connection with peers. Designers should ensure that such options are both appropriate for the intended demographic and are limited, where necessary, in the interests of user anonymity and confidentiality.

#### Strategy 4.2: Provide Supplementary Health Information

Many users initially access forums with the aim of learning about a health issue or where to find support. Consider providing up-to-date health-related information or signposting to other credible organizations. This is particularly valuable when articulating service boundaries. For example, if a forum states that it does not allow discussion of suicidal experiences, provide links to an alternative service equipped to do so to avoid users feeling dismissed without support.

### Heuristic 5: Support Informed Choices

Provide sufficient details regarding how forums operate to allow users to make informed decisions about participation and disclosure.

#### Strategy 5.1: Set Expectations for Forum Use

To support informed participation, provide information regarding what the forum intends to offer. This could include setting expectations around the type of support available and the nature of forum interactions, who the community is populated by, and the role and responsibility of forum moderators. Forums may wish to set expectations for users’ behavior on joining the forum by clearly articulating how they should interact with other users. A range of options are available for presenting this information, including as part of an initial user agreement, sign-up email, on the forum landing page, or via the use of pop-ups (bearing in mind that some people block pop-ups). A particular challenge users face is uncertainty regarding whether and when they may receive a response to their forum posts or messages to moderators. If possible, given forum resources, setting an expectation for when this may occur can be valuable (eg, “we try to respond within 24 hours”).

#### Strategy 5.2: Be Clear on Data Sharing

The ways in which forums use personal information (and why), including data privacy and confidentiality (who will see their posts and what protections users can expect), should be explicitly articulated to allow users to make informed choices regarding what they share. Users may be asked to consent to their information being processed at sign-up, where, for example, it is used for research or marketing purposes. It should be clear to forum users who exactly will see their personal information and forum posts and the implications this has for the privacy of their data. Forum designers should account for legal requirements pertaining to the region from which users access forums to ensure compliance, such as the European General Data Protection Regulation [37].

### Heuristic 6: Communicate in Autonomy-Supportive Rather Than Controlling Ways

Autonomy-supportive communication supports self-endorsed action. Its core characteristics include recognizing others’ perspectives, supporting choice and internal motivation, and avoiding coercive statements (eg, saying “you might want to consider” rather than “you must” to preface recommendations). This style of communication leads to greater decision satisfaction and adherence among health service users [38]. Forum staff should use language that supports self-endorsed action and limit directive language to only those situations where it is necessary to maintain a safe and supportive community culture. Features that scaffold this type of language use could be included in the moderator side of the forum interface, for example, as starter templates which models language use, or tips for communication style.

#### Strategy 6.1: Balance Supportive and Directive Moderation

Some forums may require posts to be edited or removed if they infringe on community rules or guidelines. Deleting posts without adequate explanation or guidance on how to post appropriately can lead to frustration and undermine rapport between users and forum staff. Moderators should attempt to communicate in a collaborative rather than admonishing tone, where possible. For example, for minor guideline violations such as posting in the wrong subforum, moderators could explain the correct way to use the forum and move the post to the correct section of the website.

If available, moderators may make use of private messaging for more extensive discussions to avoid causing the user public embarrassment, although this should be balanced against the benefit of posting such messages to an open forum and thus promoting awareness of community guidelines. Some forums use a “3-strikes” policy for minor, non–safety-related rule violations before restricting a user’s account access. This shifts responsibility for action back to the user, promoting autonomy and providing the opportunity to learn how to use the forum appropriately. However, it is important that when users act in a way that renders the community unsafe, moderators act quickly, decisively, and consistently, communicating to forum members more broadly that the community is a well-managed space.

#### Strategy 6.2: Provide Control Over Notifications

By providing control over notifications, users can manage the degree to which they are prompted to engage in forum conversations. This is appropriate given some users may find continuous and repeated exposure to forum notifications irritating or overwhelming, especially as health forums can entail emotionally charged content. However, some users also report challenges with keeping track of conversations and losing threads they had previously found helpful. Providing the option to bookmark or save interesting threads allows users to quickly find relevant information. Similarly, the ability to opt in to notifications, pop-ups, and emails can promote ease of use and ongoing engagement.

### Heuristic 7: Ensure Rewards Are Autonomy Supportive

Provide rewards that function as feedback on desired community behavior. Where such rewards are implemented, carefully consider the potential for negative consequences, such as providing an incentive for unethical behavior to “game the system” [21].

#### Strategy 7.1: Reinforce Peer Support

Consider mechanisms for providing rewards to those who engage in peer support. Examples include virtual hugs, likes, and presents, or points systems such as Reddit “upvotes.” This could be done on a more informal basis by moderators whose activities could include proactively seeking out and thanking users for particularly helpful replies to users seeking support, thus modeling the desired communication style and community culture.

#### Strategy 7.2: Recognize Community Contributors

Forums may include some method of recognition for positive community contributions for both users and moderators. These include badges that recognize rewards for “top poster” or “post of the month.” These should be co-designed with community members and caution must be exercised with regard to what is being rewarded (quantity, frequency, and impact) to ensure moderators’ and users’ authentic and autonomous motivations to help others are not hijacked by extrinsic rewards based on quantifying what is a complex human activity. Furthermore, designers should be aware that reward systems could create user hierarchies, introducing power dynamics at odds with forum goals.

### Heuristic 8: Support Mindful Attention

Design forums to facilitate sustained attention on core forum content. In recognition of the challenges of reading about and discussing potentially personal and sensitive health issues, consider prompting awareness and self-reflection on how users are experiencing the forum.

#### Strategy 8.1: Minimize Distraction

Design forums to support sustained attention on forum content. This can be achieved by presenting written information in simple, well-organized formats with minimal visual clutter and sufficient white space. Promote autonomy over interruptions to user experience, such as notifications or pop-ups, by providing options to customize or remove them. Minimizing distraction is important not only considering the emotionally sensitive nature of forum discussion but also considering that users will be approaching health forums with diverse cognitive symptoms and abilities.

#### Strategy 8.2: Prompt Self-Awareness

Prompting self-awareness of current thoughts and emotions may assist users with engaging with the forum in a way that supports their well-being. For example, some forums provide the option of completing a short-written reflection at log-in, in response to questions such as “how are you feeling right now?” Other forums may achieve a similar effect with regularly scheduled discussions, such as “check-in” threads, where users discuss how they are feeling. Guidance information, reinforced by comments from moderators, could remind users to remain vigilant to the challenges of discussing health issues online and take breaks where necessary. Some forums have recognized the value of self-reflection through exploratory writing about personal health by including features such as a diary space, where users are able to write freely without the content being accessed by other users. A private journaling space also provides an alternative for users to express themselves in relation to topics that may not be permitted for discussion on the forum.

### Principle 2: Support Competence

The psychological need for competence relates to feeling capable and effective and is similar to notions of self-efficacy. When competence is frustrated, one feels a sense of failure or helplessness [30]. Forum hosts should therefore seek to promote a sense of personal effectance and mastery over forum use and avoid design that is experienced as difficult to understand and use.

#### Heuristic 9: Apply Best Practices for Usability

Follow established usability guidelines and best practices to ensure functionality and ease of use [39].

##### Strategy 9.1: Make Forum Use Convenient, Efficient, and Simple to Understand

Designers should aim to promote convenience in forum experience and reduce barriers between users and desired actions. This could include making the sign-up process simple and efficient by reducing the number of steps involved and requesting only essential information from prospective users. Other features include automatic login to facilitate rapid forum access (optional), notification to draw attention to responses to users’ posts, and an effective search function to allow users to quickly navigate forum content.

##### Strategy 9.2: Provide Cross-Device Access

Many users access forums periodically, checking for new content or responding to recent messages, perhaps while logged in with several different devices simultaneously. Providing streamlined access to multiple devices is therefore likely to promote engagement.

### Heuristic 10: Communicate Guidelines and Protocols

Provide guidance on how to use the system and on any policies or protocols that determine how users are expected to interact.

#### Strategy 10.1: Provide Detailed Guidance on Forum Use

Support user competence by providing clear and engaging guidance on how they can use the platform effectively. This could be done as part of initial onboarding and reinforced via a visible help menu. This is particularly valuable for new users, who may be put off by complex or confusing interfaces. Topics forum designers may wish to cover include (1) core functions including how to navigate the forum, start threads, post responses, and use other features such as private messaging, where available; (2) community rules, such as restricted topics, as well as actions moderators may take if rules are broken; (3) how to engage in supportive communication with other users. This may include how to request additional support from moderators if a user is concerned about content they read on the platform; and (4) details on the confidentiality of user information and implications of anonymity for forum use, if applicable.

#### Strategy 10.2: Promote Guidance Visibility

Information regarding how a service operates, such as its terms and conditions, is not routinely read by many internet users. Designers should therefore consider how to promote engagement with forum guidance. This may include a series of short, engaging animations or videos that new users must click through before accessing the forum, a “community hub” or “how-to” section containing accessible information on how the forum works, or a pop-up that details forum rules and an agreement to abide by them, before users are able to post their first message to the forum.

### Principle 3: Support Relatedness

The psychological need for relatedness encompasses the “experience of warmth, bonding, and care and is satisfied when one feels connected to significant others.” [30]. It is likely to be promoted when forum users engage in reciprocal and mutually beneficial exchange of health experiences and social support [40].

#### Heuristic 11: Support a Sense of Meaningful Connection to Others

Support experiences of warm connection and closeness to others.

##### Strategy 11.1: Make Community Activity Salient

Individuals with health difficulties often feel isolated, so providing cues that signal belonging to a larger community can help. Relatedly, forums require an active community to generate new content and maintain momentum. Therefore, to promote the feeling that users are engaging with others in an active community, forums could show the number of users online or active in the forum, or other forum activity such as recent posts. Forum profiles could also show whether a user is online and active, or when they last posted, which may encourage others to engage in conversation. Any signal revealing user status should be customizable (ie, users may not want to signal their active status and should have the choice). Where it is not appropriate to show live users (eg, the community is small and technical limitations), other low-tech and static representations of community members can also serve this function (eg, collections of anonymized avatars, featured member profiles or stories, a world map marked with points showing where different members are from, etc).

##### Strategy 11.2: Consider Synchronous Communication Options

Supplementary, synchronous communication features can provide the opportunity for direct engagement with others, supporting a sense of communal presence and interactivity. Examples include prescheduled “chats” or “question and answer” sessions hosted by forum staff using live chat or video-streaming software. Such events could be themed around topics chosen by the community to promote engagement and peer interaction. Some forums also provide users with the ability to interact with staff and other users via instantaneous direct messaging, creating a space for private conversations. Designers should, however, consider potential challenges such as privacy issues, service misuse, and increased demand on moderators.

##### Strategy 11.3: Support Meaningful Moderator Interactions

Forming meaningful connections and helping others within a forum environment involve communicating in ways that are welcoming, express empathy, and provide relevant information and advice. However, showing understanding and providing emotional support by text is a highly skilled task. Supporting moderators in learning and exercising these skills can help support this connection-making. This can form part of moderator training and support, and scaffolding can also be built into the system, for example, through the provision of templates, examples, or tips. To further enhance peer engagement, some forums employ staff or volunteers with lived experience in “supporter” roles, whose responsibilities may have less of a focus on rule enforcement but rather on interacting with forum users, generating opportunities for meaningful peer connection.

##### Strategy 11.4: Optimize Experiential Groupings

Health forums usually group users by health condition, which is intended to engender a sense of community. Designers should consider how the experiential grouping around which communities are formed may impact perceived relatedness. For example, it is feasible that a platform for young people which includes both those in their early teenage years and young adults may lead to relatedness frustration if one particular age group predominates and users in the minority are unable to relate to the challenges expressed by a majority of forum users. Other forums use intentionally broad inclusion criteria to ensure that forums are well populated and include diverse experiences. Designers may wish to consult current or potential users to achieve an appropriate experiential grouping in light of the forum’s goal. Subforums can also be used to funnel users to those with more specific shared experiences.

##### Strategy 11.5: Consider Nontextual Interaction Options

Some users value nontextual interaction options such as emojis, images, or GIFs for their ability to easily communicate ideas and emotions. Other features, such as the ability to “like” a comment, provide an easy way for users to indicate that another’s post has been helpful. Views on these features are mixed; some see them as essential, while others view them as a distraction from the intended text-based nature of online forum communication. Designers should work with their users to determine the best approach for their context and consider allowing users to disable images (especially moving imagery such as animated GIFs) if these are permitted.

##### Strategy 11.6: Consider “Off-Topic” Threads

Some forums include “off-topic” discussions not explicitly related to health issues, such as general interests or hobbies. These threads can be popular and provide a place for relationships to develop. Such threads should, however, align with the wider aims and tone of the service.

### Heuristic 12: Support a Sense of Belonging

Consider how forums can communicate the sense that users are both welcome in the community and part of a shared space with like-minded individuals.

#### Strategy 12.1: Make Community Membership Salient

Promote a sense of belonging by emphasizing that users are members of an online group by, for example, referring to the service as a community with members, rather than a forum with users. Promote community ownership through engagement opportunities such as asking users to suggest themed content, provide feedback on what could be improved, or participate in service development sessions.

#### Strategy 12.2: Encourage Engagement by New Users

Some forums have dedicated spaces for new users to introduce themselves and initiate peer interactions. Some also have dedicated “welcomers,” who are moderators or members of the community assigned the responsibility of acknowledging and inducting new users. The intention here is to ensure that users have a friendly, welcoming, and rapid initial interaction which promotes a positive impression of the forum, increasing the likelihood of ongoing use.

### Heuristic 13: Support Caring for Others

Design forums to provide opportunities for mutual support and reinforce caring behaviors.

#### Strategy 13.1: Encourage Supportive Contributions

Forum designers should consider how to encourage supportive communication while minimizing stigmatizing perspectives and online toxicity. Practical steps toward such a culture include clear guidance on sign-up encouraging users to share their views, support others, and note when they have found another user’s post helpful. These behaviors can also be modeled and reinforced by moderators, whose style of communication sets the tone for the wider community. Some forums seek to generate supportive communications with subsections or threads themed around requests for support, such as “I need help with...” threads. Consider ways to show or give care in virtual form, such as alternatives to the “like” button now offered by services such as LinkedIn, which may include, for example, a hand holding a heart, the “recovery” emoji (a heart wrapped in a bandage), or the hug emoji, all of which are commonly used for showing sympathy.

#### Strategy 13.2: Make Positive Impact Salient

Receiving evidence that one’s behavior has positively influenced others can promote relatedness [18], while also promoting autonomy and competence. Perhaps the most powerful reward or acknowledgment for a moderator’s efforts (or the efforts of community members trying to help others) is evidence of their own positive impact, and such evidence can help support resilience over burnout [41]. Forums may therefore seek to include mechanisms for convenient expressions of gratitude, such as a mechanism to recognize another user’s comment via a button communicating “thanks,” “this really helped,” or “I appreciate this.” Moderators could also be guided to proactively respond to users directly, expressing appreciation for their positive contributions to forum conversations. For moderators specifically, providing aggregated data about how many users viewed their responses or clicked on links that they provided could be a powerful way to show impact.

### Principle 4: Support Safety

User safety is a primary concern for health services, aligning with the principle that interventions should first do no harm. The inclusion of the principle to support user safety is grounded in the assumption that users who perceive a forum to be a supportive and well-managed place, where rule breaking and distressing content is minimized, will be more likely to read and post to the forum [6]. Safety relates to not only the minimization of harm but also psychological safety, or the perception by forum members that they feel able to share what are often very personal health experiences, with the confidence that responses will be respectful and constructive.

#### Heuristic 14: Establish Community Guidelines

Establish clear rules designed to promote peer support and discourage behaviors likely to undermine well-being.

##### Strategy 14.1: Balance Openness and Risk

Rules should facilitate supportive and open discussion of health-related issues, yet restrict topics likely to cause harm to the community. The challenge for designers lies in disallowing content that could reasonably be expected to cause psychological distress or lead to physical harm to those who read forum discussions, while avoiding the space becoming overly sanitized such that frank discussions of health experiences cannot occur. Specific considerations include whether users can discuss sensitive topics such as suicidality and self-harm, eating behaviors, advice regarding medical treatments, and misinformation. To retain focus on health, some forums may restrict debate on potentially controversial topics, such as topical social issues or politics. Such rules should be carefully considered with reference to the focus and demographics of the forum, and designers may wish to work with users and/or experts by experience to promote the acceptability of community guidelines.

##### Strategy 14.2: Implement Timely Rule Enforcement

Minimizing the risk of distress related to forum content relies on its timely recognition and removal. Forum moderators could be recruited to work in shifts to ensure that there is always someone available to manage safety issues as they emerge. If this is not possible, users should be notified if moderation is limited to specific hours. Some forums implement premoderation, where forum posts are manually reviewed by content moderators before going live on the forum. This thorough approach significantly reduces the risk of inappropriate content but limits synchronicity in user-to-user interaction and requires significant resources. Important considerations regarding timely moderation include daily and seasonal changes in the volume of forum posts (eg, increases in forum use during periods when people may be more likely to seek support, such as examination periods or public holidays) and the moderation team’s capacity to deal with spikes in workload. Moderation teams should be appropriately resourced to meet such demands to ensure continuity of service and reduce the risk of staff burnout.

##### Strategy 14.3: Use Technology to Facilitate Moderation

Evolving technical approaches allow the automated detection of problematic posts or the triaging of posts for moderation, showing the potential for technical systems to support moderators and reduce workload [42]. Automatic word-blocking programs can be trained by humans to identify and remove posts that include prohibited words. Other automated approaches involve triage systems that highlight posts based on content (eg, suicidal ideation) or time (posts that have not received a reply within a particular time frame), allowing moderators to efficiently respond to those most urgently requiring support. Some forum environments allow users to flag problematic content themselves via a user-report system, which is likely to support the efficient identification and management of rule-breaking or concerning content. Given the complexity of determining whether an issue may require more focused attention by moderators, such systems should be rigorously tested to avoid unintended outcomes.

### Heuristic 15: Establish Procedures for Managing Distress

Discussions of health-related issues hold inherent potential to cause distress. Designers should consider how to manage distress within the constraints of the online forum context.

#### Strategy 15.1: Use a Stepped Approach to Managing Distress

A stepped or graded approach to user support is valuable for forum staff because it defines how to respond in specific scenarios. For example, a forum might operationalize “low,” “medium,” and “high” levels of concern regarding a user’s well-being. A low concern situation may warrant an empathetic response; a medium concern may require the moderation team to offer more focused support, for example, through direct message or signposting; and a high degree of concern may involve users feeling unsafe, requiring staff to consult specific risk management protocols. Such a protocol should define when specific responses, such as providing a link to a suicide hotline or offering a call from a mental health professional, are warranted. These actions will be influenced by factors such as the duty of care an organization holds over forum users and the amount of information the organization has about each user. It is important for both moderator and user safety that such protocols are clearly defined and reviewed regularly.

#### Strategy 15.2: Provide Comprehensive Moderator Support

Moderation can be demanding and often involves intense interpersonal interactions focused on distressing topics. Designers should consider how to ensure that forum moderators feel confident and supported in their roles. Strategies include both initial and ongoing training in the forum’s subject matter and in providing support to forum users; supervision from experienced colleagues to discuss challenges and personal well-being; peer-to-peer support between moderators, which is particularly valued during shifts to facilitate shared decision-making; and postshift debriefing [7]. It is important that volunteer moderators are not limited in accessing appropriate support services due to their role as unpaid staff.

### Heuristic 16: Consider the Limits and Impacts of Anonymity and Confidentiality

The ability to remain anonymous and the confidentiality of personal information are likely to influence users’ willingness to engage with online forums. They also influence the extent to which safeguarding concerns can be managed.

#### Strategy 16.1: Balance Anonymity and Confidentiality With Safeguarding Responsibilities

Some forums require only an email at sign-up, while others request more extensive personal information including demographics and location. This influences the ability of forum staff to follow up with a user if a safeguarding issue emerges. It should be noted that many forum users access them precisely because they can be anonymous, and their posts are untraceable back to their offline identity. This allows them to speak more freely, without fear of being judged or receiving unwanted offline input. Forum designers should consider how to balance the advantages and possible disadvantages of user anonymity, bearing in mind their organizational responsibility and duty of care to users. If forums do hold confidential personal information, conditions under which those details will be shared, for example, with emergency services, should be made explicit.

### Heuristic 17: Manage Content Visibility

Consider how forums can be designed to provide control over which content users see. This can help to promote informed choices around exposure to issues users may find distressing.

#### Strategy 17.1: Content Structure, Tags, and Warnings

Forums can be designed to help users understand whether forum content may be distressing before they access it. This could be achieved via subforums, with dedicated areas for discussing potentially distressing topics. Some forums also implement content warnings or topic tags, which inform users of the likely distressing nature of the thread before they click on it. Similarly, within threads, some forums have a “spoiler” function. This blanks out some or part of a user’s post and requires a reader to manually click to reveal the text.

### Heuristic 18: Establish Procedures for Managing Problematic Forum Use

Anticipate and plan to address problematic behaviors that could undermine the safety and effectiveness of online health forums.

#### Strategy 18.1: Develop Protocols for Addressing Forum-Specific Problematic Behavior

Develop plans for managing specific problematic behaviors common to online forums: (1) trolling: users engaging in intentionally offensive or provocative behavior such as posting inappropriate content or instigating inflammatory discussions; (2) inappropriate interactions: users engaging in forum use that is incongruent with the forum’s aims and rules, for example, starting conversations not relevant to the forum or using private messaging to request personal information; (3) arguments: users engaging in discussions that go beyond mere disagreement to personal insult or threats. Rules that clearly articulate the line between disagreement and rule-breaking disputes help inform both users and moderators about what is acceptable; (4) multiple account creation: users who have had their accounts restricted creating further accounts to continue with their problematic behavior; and (5) spam: irrelevant or inappropriate content usually intended to disrupt the forum’s function.

## Discussion

### Overview of Principal Findings

This paper reports SDT-informed technology design guidance adapted for the context of online health forums. SDT is a major theory of well-being, and designing to support basic psychological needs has been proposed to promote well-being in technology use [16]. Drawing on an existing hierarchical SDT framework [18], we presented heuristics in support of user autonomy to support forum use in ways that align with users’ self-endorsed behavior, goals, and values. Competence-supportive heuristics were modified to account for the importance of ease of use in user experience. For supporting relatedness, a key concern for forum designers, we adapted heuristics to articulate how forums can be designed for meaningful and supportive interactions. Finally, safety-focused heuristics detail essential issues forum designers should consider when developing approaches to moderation, rules, and user distress.

### Integration With Existing Literature

This work builds on challenges in forum implementation identified in prior research, including the difficulty of balancing conversational openness with regulation [6]. A forum supporting young people experiencing suicidal ideation implemented robust safety measures including collecting emergency contact information and automatically blocking suicide-related conversations [43]. Some users appreciated these precautions for the reassurance they offered, yet others felt that restricting discussions perpetuated stigma and discouraged sharing their experiences [44]. This tension further highlights the value of ongoing stakeholder engagement to inform forum design and adaptation but reinforces the reality that these perspectives may sit in tension. Relatedly, research into moderators’ perspectives reveals how implementing rules is often more nuanced than surface interpretations of forum guidance suggest [45], with challenges including indirect references to risk, time pressures on decision-making, and the emergence of unforeseen safeguarding issues for which protocols do not exist. This underlines the need for continuous monitoring and adjustment in each forum’s implementation, with particular attention paid to the expertise and community knowledge of forum staff.

This work adds to literature using SDT in intervention design. A recent review highlighted how behavior change interventions promote engagement using design features that overlap significantly with our recommendations [46], including intuitive interfaces, customizable features, feedback, and human encouragement. Organizations may also benefit from evaluating their forums using SDT-informed measures of psychological need satisfaction in technology use [47]. Beyond SDT, user-centered design principles stress the importance of treatment efficiency, ensuring that users can quickly address their relevant goals, and learnability, helping users easily understand and navigate the platform [48]. Beyond focusing on specific implementation techniques, designers may benefit from taking a holistic view of their services, understanding how various components of their digital offer combine to form a coherent “intervention system” [49]. Part of this work may involve ongoing iteration of a logical model or program theory to understand the gap between intended and actual impacts. Aligned with our recommendation to refine forums in collaboration with users to gain insights into their goals, values, and preferences, contemporary intervention design guidelines emphasize the value of stakeholder involvement throughout this theory-development process [17].

### Strengths and Limitations

This study is the first to draw on broad experiences and psychological theory to generate forum design guidance, extending SDT to a novel domain. However, there are several notable limitations. This work reflects a UK-based team with a primary area of focus on mental health, which may limit transferability to other settings. Forums are likely to be accessed by people with diverse cognitive and physical abilities and our recruitment process did not engage participants on this basis. Future research centered on understanding the experiences of people with various health challenges could enrich findings in important ways.

This work is also necessarily managerial in tone, aimed at those developing or implementing forums, and may have recruited a sample biased in favor of more engaged staff and users. This may have obscured tensions in perspectives on forum design between, for example, forum moderators and users with negative experiences of current services. Given our approach is rooted in existing practice, future research could explore more radical approaches to user empowerment, such as participatory governance models.

SDT has significant empirical support but is by its nature broad, and more focused psychological theories, such as those relevant to particular health issues, may be more directly relevant or valuably complement SDT-informed guidance for specific forums. Furthermore, an important direction for future research is the direct evaluation of theory-based online health forum interventions across outcomes, such as well-being, engagement, and safety, to fully assess the usefulness and cost-effectiveness of conceptually informed interventions.

### Conclusions

Combining a consideration for safety with an explicit focus on autonomy, competence, and relatedness, we have presented forum designers and organizations with theory-informed guidance for service development. While this approach aligns with an extant body of research linking needs satisfaction with positive technology-related outcomes, the growing popularity of online health forums warrants renewed focus on how these widely used services can be optimized using theory and evidence to the ultimate benefit of user well-being.

## Supplementary material

10.2196/85411 Multimedia Appendix 1 Participant demographics and topic guides.

10.2196/85411 Multimedia Appendix 2 Illustrative stakeholder perspectives.

## Abbreviations

iPOF: Improving Peer Online Forums
SDT: self-determination theory
