# Supplementary material for: Design Guidelines for Online Health Forums: User-Centered Design Approach
Source: J Med Internet Res. 2026 Sep 14;28:e85411. doi: 10.2196/85411 (PMC13574306; doi:10.2196/85411)
Supplement: Supplementary file 1 [file jmir-v28-e85411-s001.docx]

**Multimedia Appendix 1**

**Staff interview demographics**

| **Role and participant number** | **Age** | **Gender** | **Ethnicity** |
| --- | --- | --- | --- |
| Host 1 (Senior community manager) | 46-55 | Male | White/White British |
| Moderator 1 | 16-25 | Female | White/White British |
| Moderator 2 | 16-25 | Female | White/White British |
| Moderator 3 | 26-35 | Male | White/White British |
| Moderator 4 | 36-45 | Female | Black/African/Caribbean/Black British |
| Moderator 5 | 16-25 | Female | Asian/Asian British |
| Host 2 (Senior community manager) | 46-55 | Female | Other Ethnic Group |
| Moderator 6 | 26-35 | Female | White/White British |
| Host 3 (Senior community manager) | 56-65 | Female | White/White British |
| Moderator 7 | 26-35 | Female | White/White British |
| Moderator 8 | 26-35 | Female | White/White British |
| Host 4 (clinical academic) | 56-65 | Male | White/White British |
| Moderator 9 | 26-35 | Male | White/White British |
| Moderator 10 | 26-35 | Female | White/White British |
| Moderator 11 | 26-35 | Female | Mixed/Multiple Ethnic Groups |
| Moderator 12 | 26-35 | Female | White/White British |
| Host 5 (Clinical academic) | 46-55 | Female | White/White British |
| Moderator 13 | 26-35 | Female | White/White British |

**Staff interview topic guide**

*Topic 1: Details about the participant and their role in relation to forums*

- Can you tell me a bit about your role?
- Are you [hosting/moderating/researching] a specific type of forum?
  - target users
  - design
  - role of moderators

*Topic 2 – Pattern of role*

- How did you come to have this role?
- How long have you been in this role?
- How much time do you spend in this role?
- how has pattern of use changed over time? why?
- if left – why left?

*Topic 3: Sharing experiences*

- Can you describe what you think happens in peer online mental health forums [or specifically the one they are involved with]?
  - what kinds of things are discussed? how? why? by who?
  - What do you see as the role of a forum?
  - What do you like about forums? Anything you don’t like or wish was different?

*Topic 4: Outcomes/impacts*

- Why do you think some NHS Trusts / charities offer peer online mental health forums?
  - what impact might it have on their organisation? (explore positives and negatives)
  - why? and how? (explore mechanism of all impacts and contextual factors that make this more or less likely to occur)
- What do you think people can gain from being in a forum? Explore positive and negative impacts.
- For each impact identified
  - how do you think this happens? (explore underlying mechanism and any contextual factors that allow this to happen)
  - what things make it more likely to happen? what might prevent this happening? (explore triggering contextual factors)
  - are there any particular kinds of people more likely to experience (each impact)? Or any kinds of people who might not get this? Why?

*Topic 5: engagement*

- What do you think engages people to use forums?
  - what might trigger someone to post?
  - why do some people use them a lot and others only once or twice?
- Why do you think some people observe but don’t post?
  - what do they get out of the forum?
- Why do some people become “superusers”?
  - what do you think might be the impacts of that? how? why and in what context?
- Why do you think some people become forum moderators?
  - what do you think might be the impacts of that? How? why and in what context?

*Topic 6: Designing best policy and practice. The aim of the study is to develop some best policy and practice tools. These might include 1) how to design a forum; 2) training and support for moderators; 3) information for commissioners and health professionals about why peer online mental health forums are needed*

- What advice would you give to someone starting to design a peer online mental health forum – explore in depth and why i.e. mechanisms?
- What kind of training do you think moderators need?- explore in depth and why ie mechanisms? and how?
- what kind of ongoing support do moderators need? explore in depth- why ie mechanisms? and how?
- What information do you think commissioners and health professionals need about forums to invest their time / money?
- Ask if anything they would like to add to help us understand the impacts of peer online mental health forums

**Forum user interview demographics**

Topic 1 – Mental health self-efficacy

*Exploratory questions:*

- Could you describe how using the forum influences your mental health?

*Theory-informed questions:*

- According to some of the research we have looked at, one of the ways forums can be helpful is by giving people convenient access to information about how they can manage their mental health. What do you think of that idea?
- How do you know whether to trust the information and advice you find on mental health forums?
- Does the fact that some people share advice based on their own personal experience, influence how you view their advice?

Topic 2 – Psychological safety

*Exploratory questions:*

- We’re interested in what makes a forum feel like a safe space. What makes a forum safe enough for you to discuss and read about issues like mental health?

*Theory-informed questions:*

- Do forum rules influence how safe the forum feels?
  - Are there any specific rules/examples that come to mind?
- Do moderators influence how safe the forum feels?
  - Can you think of any examples of things moderators do to promote safety?
- Some research shows that people might come across things on forums that they find upsetting. Have you ever experienced that?
  - How did you react?
  - Did that influence how much you used the forum
- One idea from previous research is that people tend to stay on forums when they feel welcomed into the online group. What is your view of that idea?
  - What influenced how welcoming the forum felt when you joined?
  - Have you ever used a forum that felt unwelcoming? What was your reaction?
- Some forums have ways to highlight that posts are about sensitive topics, for example there may be sub-forums dedicated to sensitive issues, tags on posts, or trigger warnings. Does the forum have those features?
  - Does this influence how safe the forum feels?
- Some previous research suggest that online forum conversations could encourage behaviours that could be harmful, such as self-harm or restrictive eating. Is that something you have come across online?
  - What was your reaction?
  - how is that managed by the forum/organisation?
- Some people report that they started to post in online forums because they saw other people getting friendly and helpful responses. What is your view of that?

Topic 3 – Forum access and resource use

*Exploratory questions:*

Have forums influenced your views on other types of mental health support?

*Theory-informed questions:*

Has your use of the forum led to changes in the way that you use of other types of mental health support?

Topic 4 – Forum moderation

*Exploratory questions:*

- We’re interested in how moderators impact on forum users’ experiences. What is your understanding of the role of the moderators on the forums you use?
- From your perspective, what is good forum moderation?
  - Can you think of an example where you’ve seen good forum moderation?
- Thinking specifically about moderators’ responses to forum posts – what are the characteristics of helpful moderator responses?
  - How do you tend to react to these kinds of responses?
- And what are the characteristics of unhelpful moderator responses?
  - How do those kinds of posts impact you?

*Theory-informed questions:*

- I would like to get your thoughts on ideas from previous research about what a helpful moderator response might be like. How important is it that a moderator responds in the following ways:
  - Quickly
    - is it possible for moderators to respond too quickly?
  - Responds to the specific issues you have raised
  - Has a warm tone
  - Is empathetic
  - Is there anything else we have missed?
- A bad response could have the opposite characteristics. For example, a bad response might ignore or downplay your problem which is likely to make you feel disconnected from the people on the forum and maybe even less likely to post again. What is your view on that?
  - Have you seen this happen?
  - How did you/the other user react?
- Has a moderator ever restricted your ability to talk about something on the forum
- Can you think of an example?
- How did you feel about this?
- What impact did that have on your forum use?

Topic 5 – Social support

*Exploratory questions:*

- How does connecting with other people online affect your wellbeing?
- Does the forum influence your sense of being supported by other people?

*Theory-informed questions:*

- One thing we want to understand is what it is like for people to read posts that resonate with their experience. For example, someone may post about how they have been through the same kinds of difficulties or situations as you. How do you respond to reading those kinds of posts?
  - There’s a theory that by reading posts like that, people can see that others are going through similar things, which may make people feel less isolated with their experiences, and potentially feel less stigma, too. What’s your opinion on that?
- We’re interested in the kinds of responses people get in online forums, and in particular, what a helpful response from other forum users looks like. When you post to the forum, what kinds of responses do you typically want from other users?
  - How important is the speed of response from other users?
  - For a response to seem helpful, some research says that it is important for users who reply to stay on topic, and directly address the original poster’s concerns. What do you think?
  - A warm and empathetic tone also seems to be important for some people when they initially post to the forum. What’s your view on that?
- To what extent do you feel a sense of connection with the online community on the forum?
  - Has this changed over time? If so, why?
  - Have you ever used forums where that sense of connection has been different? If so, why?
- One of the ideas we have seen in previous research is that connecting with others online may provide forum users with a feeling of belonging. Is that true in your experience?
- Do you tend to respond to other people who are looking for support on the forum?
  - How does it feel to offer that support?
  - The reason I ask is that some people find it empowering to be able to draw on their own experiences to help others. What’s your view on that?
- Has using the forum influenced the way you feel about asking for support from the people around you?
- How do you decide when and how much of your own personal experiences to share when posting to the forum?
  - Do you find that you are more likely to share your experiences when other people post about their own experiences? If so, why?

**Topic 6 – Forum design**

*Exploratory questions:*

- Can you describe the main features of the forum website? How do they impact your experience of the forum?

*Theory-informed questions:*

Two factors that seem important are anonymity and accessibility.

- How does the ability to post anonymously influence the way you use the forum?
  - Are there any other positives or negatives that you can think of?
    - Could anonymity make some people more likely to break the rules?
- Previous research suggests that one of the potential advantages of a forum is that they are usually available whenever people need them – what is your view on that?
- Does the forum you use have emojis or the ability to like posts?
  - Do you use them?
  - Why/why not?
- Does the forum have a search function?
  - Do you use it – and how?
  - (If no) would that be useful?

**Forum user interview topic guide**

| **Forum code_participant number** | **Age group** | **Gender** | **Ethnic group** |
| --- | --- | --- | --- |
| Chaffinch_001 | 16 - 25 | Female | Black/African/Caribbean/Black British |
| Chaffinch_009 | 16-25 | Prefer not to say | Prefer not to say |
| Chaffinch_022 | 26-35 | Female | White/White British |
| Dunnock_002 | 16-25 | Female | Prefer not to say |
| Dunnock_003 | 16 - 25 | Female | White/White British |
| Dunnock_043 | 16 - 25 | Female | White/White British |
| Dunnock_046 | 16 - 25 | Female | White/White British |
| Dunnock_073 | 16 - 25 | Prefer not to say | White/White British |
| Dunnock_090 | 36 - 45 | Female | White/White British |
| Dunnock_196 | 26-35 | Female | White/White British |
| Dunnock_229 | 16-25 | Male | White/White British |
| Dunnock_458 | 16-25 | Prefer to self-degine (Transgender male) | White/White British |
| Dunnock_646 | 36-45 | Female | White/White British |
| Goldfinch_001 | 46 - 55 | Female | White/White British |
| Jay_001 | 16 - 25 | Male | Black/African/Caribbean/Black British |
| Magpie_022 | 76-85 | Female | White/White British |
| Magpie_032 | 26 - 35 | Female | White/White British |
| Magpie_073 | 56-65 | Female | White/White British |
| Magpie_077 | 56 - 65 | Female | White/White British |
| Magpie_100 | 55-65 | Female | White/White British |
| Magpie_126 | 46-55 | Female | White/White British |
| Magpie_178 | 26-35 | Female | White/White British |
| Magpie_213 | 26-35 | Female | White/White British |
| Magpie_214 | 66-75 | Female | White/White British |
| Magpie_304 | 56-65 | Female | White/White British |
| Magpie_321 | 46-55 | Female | Mixed/Multiple Ethnic groups |
| Magpie_326 | 56-65 | Female | White/White British |
| Magpie_342 | 56-65 | Female | White/White British |
| Sparrow.2_064 | 36-45 | Female | White/White British |
| Sparrow.2_119 | 56-65 | Male | White/White British |
| Sparrow.8_052 | 26-35 | Female | White/White British |
| Sparrow.8_072 | 26-35 | Female | Other ethnic group |
| Sparrow2_002 | 56 - 65 | Female | White/White British |
| Sparrow2_003 | 66 - 75 | Female | White/White British |
| Sparrow2_006 | 46 - 55 | Female | White/White British |
| Sparrow2_011 | 36-45 | Female | White/White British |
