# Supplementary material for: Design Guidelines for Online Health Forums: User-Centered Design Approach
Source: J Med Internet Res. 2026 Sep 14;28:e85411. doi: 10.2196/85411 (PMC13574306; doi:10.2196/85411)
Supplement: Supplementary file 2 [file jmir-v28-e85411-s002.docx]

# Multimedia Appendix 2: Illustrative Stakeholder Perspectives

## Principle 1: Support Autonomy

| **Heuristic** | **Illustrative Quote** | **Source** |
| --- | --- | --- |
| **H1: Apply best practice for accessibility** | I think you need to have some kind of basic IT skills level to access the forum. It's not as simplistic as it could be. It's a lot of words, it's very busy, there's no nice pictures. I think it could be when you log in it should be like, 'Welcome...' then 'What category would you like to look at?' | Robin_009, forum user (interview) |
| **H2: Empathize with your user's frame of reference** | I still speak to them [moderators] now actually and I think what I really like about them is they don't just care about what is wrong, they care about getting to know me personally as well. I’ve been speaking to him [moderator] now for a year and he still remembers details I mentioned on day one. | Dunnock_046, forum user (interview) |
| **H3: Provide meaningful rationale** | Some people find some of the rules difficult but being able to explain, explaining in detail... I've maybe only had a handful of people been abusive to me... having that open communication where people can feed back and to explain things properly has been helpful | Goldfinch_001, forum moderator (interview) |
| **H4: Provide meaningful choices** | Offering choice: profiles, avatars, email preferences, reactions. Ways to make personal identity salient. | Workshop 1, autonomy (field notes) |
| **H5: Support informed choices** | Consulting with the user group e.g. on potentially controversial issues (e.g. to what extent people can talk about diagnosis, medication). It was noted that sometimes what’s best for the community isn’t what will come out on top in a vote – need for moderator expertise in decision making. | Workshop 1, autonomy (field notes) |
| **H6: Communicate in autonomy supportive rather than controlling ways** | A ‘3 strikes’ rule could help users feel like they're given responsibility to take action. Reiterating rules and giving warnings may have the same effect – less autonomy infringing than simply deleting posts or banning people – a chance to get the issue out, have it recognised and responded to. | Workshop 1, autonomy (field notes) |
| **H7: Ensure rewards are autonomy supportive** | Delegate power to users through voluntary roles e.g. 'welcomers', which gives ownership and status. Similarly, badges that reflect effort and reinforce desired action (superuser badge, ‘asthma captain’, birthday ambassador etc). | Workshop 1, autonomy (field notes) |
| **H8: Support mindful attention** | Have a way to track/subscribe to posts. | Workshop 1, autonomy (field notes) |

## Principle 2: Support Competence

| **Heuristic** | **Illustrative Quote** | **Source** |
| --- | --- | --- |
| **H9: Apply best practices for usability** | I've tried to join online forums but you have to set up an account and send in your email address and there's quite a few steps. So I've never actually managed to get through the process of setting up an account. I've always just thought, 'Oh god I can't be bothered doing all that.' | Starling_001, forum user (interview) |
| **H10: Communicate guidelines and protocols** | Our community guidelines is something that's ever evolving. It has to be... a lot of our young people are very savvy about for example AI and keywords... so they will constantly find ways of talking about topics or trying to talk about topics that don't meet our boundaries so it is something that's very much a live document and constantly updated. | Dunnock_001, moderator (interview) |

## Principle 3: Support Relatedness

| **Heuristic** | **Illustrative Quote** | **Source** |
| --- | --- | --- |
| **H11: Support a sense of meaningful connection to others** | …humanising moderators so that they’re trusted and there’s less of an ‘us and them’ divide. Things help like: introduction posts, profiles with personal details, avatars, offering opinions. Dunnock even used AI generated pictures, so moderators seem like real people. | Workshop 1, autonomy (field notes) |
| **H12: Support a sense of belonging** | Importance of the ‘onboarding’ experience in helping users feel connected. E.g Chaffinch have a dedicated ‘introduce yourself’ section which moderators and other users are very active in. Messages are friendly and invite further conversation…some forums have lived experience roles to promote engagement and access to lived experience perspectives…helps users feel connected and part of a lively community. | Workshop 3, relatedness (field notes) |
| **H13: Support caring for others** | Specific threads e.g. “I need a hug” – very popular and needs very careful moderation. | Workshop 3, relatedness (field notes) |

## Principle 4: Support Safety

| **Heuristic** | **Illustrative Quote** | **Source** |
| --- | --- | --- |
| **H14: Establish community guidelines** | Balancing openness against restricting content – a key issue for user autonomy and safety. Having a ‘stepped’ approach to risk and corresponding action can be helpful and clarifies how moderators should respond. | Workshop 4, safety (field notes) |
| **H15: Establish procedures for managing distress** | Moderators need to be able to raise challenges in their role in a way that feels safe and accessible eg. via WhatsApp, a reporting feature, and supervisor debrief. This is very much about organisational culture. | Workshop 3, relatedness (field notes) |
| **H15: Establish procedures for managing distress** | Burnout can occur if there are too few moderators. Someone needs to be able to step in if things become too much. 'Diffusion' of responsibility, and the ability to draw on support of colleagues, are important. | Workshop 1, autonomy (field notes) |
| **H16: Consider the limits and impacts of anonymity and confidentiality** | Anonymity was a really powerful thing because it allows people to talk about things that are often quite taboo... young people who are concerned about what the fallout might be of what they are saying can actually say it safely and knowing that it's not going to trigger an immediate safeguarding response…we do get a lot of young people that for example self-harm and if they told the teacher then that would immediately go back to their parents as a safeguarding issue | Dunnock_002,moderator (interview) |
| **H17: Manage content visibility** | Adding trigger warnings to thread titles is something young people find helpful. [Chaffinch] also have a 'spoiler' tool, so it hides a section of a post that is only revealed when you press a button…community members use this in their own posts when they want to share something but worried about it triggering others. | Workshop 4, safety (field notes) |
| **H18: Establish procedures for managing problematic forum use** | Mechanism for moderators to get help in difficult situations e.g. senior moderators sign off responses in risk related scenario/where someone is banned/safeguarding. | Workshop 1, autonomy (field notes) |
